# Chemotherapy combined with Shenmai injection alleviates inflammatory response and side effects in the patients with advanced colorectal cancer: A retrospective analysis

**DOI:** 10.12669/pjms.40.9.9881

**Published:** 2024-10

**Authors:** Yude Jin, Wei Zhu, Hanbin Dai, Xiaowei He

**Affiliations:** 1 Yude Jin, Department of General Surgery, The First Affiliated Hospital of Huzhou University, 158 Guangchang Hou Road, Huzhou, Zhejiang Province, P.R. China; 2 Wei Zhu, Department of General Surgery, The First Affiliated Hospital of Huzhou University, 158 Guangchang Hou Road, Huzhou, Zhejiang Province, P.R. China; 3 Hanbin Dai, Department of General Surgery, The First Affiliated Hospital of Huzhou University, 158 Guangchang Hou Road, Huzhou, Zhejiang Province, P.R. China; 4 Xiaowei He, Department of General Surgery, The First Affiliated Hospital of Huzhou University, 158 Guangchang Hou Road, Huzhou, Zhejiang Province, P.R. China

**Keywords:** Colorectal cancer, Chemotherapy, Shenmai injection, Immune function

## Abstract

**Objective:**

To assess the inflammatory response and side effects of chemotherapy combined with Shenmai injection (SMI) in the treatment of patients with advanced colorectal cancer (CRC).

**Methods:**

This retrospective cohort study included the clinical data of 152 patients with advanced CRC admitted to the First Affiliated Hospital of Huzhou University from April 2020 to April 2023. The patients were divided into control group (patients received chemotherapy treatment, n=75) and observation group (patients received chemotherapy combined with SMI, n=77) based on the treatment received. Tumor control rate, levels of immune function indicators before and after treatment, levels of inflammatory factor indicators, and incidence of toxic side effects in two groups were analyzed.

**Results:**

Tumor control rate in the observation group (89.61%) was higher than that in the control group (77.33%) (*P*<0.05). After the treatment, the levels of CD3^+^, CD4^+^, CD4^+^/CD8^+^in both groups were significantly higher than before the treatment, and significantly higher in the observation group compared to the control group (*P*<0.05). After the treatment, serum levels of interleukin 6 (IL-6), tumor necrosis factor-alpha (TNF-α), and interleukin-8 (IL-8) in both groups decreased compared to pretreatment levels, and was significantly lower in the observation group (*P*<0.05). The incidence of adverse reactions in the observation group was significantly lower than that in the control group (*P*<0.05).

**Conclusions:**

Compared with chemotherapy alone, chemotherapy combined with SMI better alleviates inflammatory response in patients with advanced CRC, enhance immune function, and improve tumor control rate, with a lower incidence of toxic side effects.

## INTRODUCTION

Colorectal cancer (CRC) is characterized by multiple malignant tumors in the digestive system.^1^ In recent years, with the dietary and lifestyle changes, incidence of CRC has continued to increase, and it has become a type of malignant tumor that seriously threatens people’s quality of life.^1^,^2^ In most patients, CRC manifests by varying degrees of decreased appetite, bloody stools, and abdominal pain. Since these symptoms lack specificity, the diagnosis of CRC is often made in the late stage of the disease, making treatment and good prognosis difficult.^3^,^4^

Currently, chemotherapy is a main method of treatment of patients with advanced CRC, and can inhibit lesion progression and prolong the survival.^5^ However, chemotherapy is also associated with varying degrees of damage to normal cells around the lesion. Moreover, the toxic side effects of chemotherapy drugs can make it difficult for patients to fully tolerate them, thereby affecting the treatment process.^6^

In recent years, the application of traditional Chinese medicine (TCM) in the treatment of cancers has been on the rise.^7^ From the perspective of TCM, patients with cancer often have a deficiency of qi, and TCM believes that chemotherapy will further exacerbate blood stasis, weaken the functions of spleen, kidney, and liver, and imbalance the yin and yang five elements.^8^ Therefore, the pathogenesis of the disease lies in the disorder of qi deficiency, imbalance of yin and yang, and positive deficiency with toxin and blood stasis.^7^,^8^ Shenmai injection (SMI) is a commonly used traditional Chinese medicine preparation in clinical practice, containing Ophiopogon japonicus and Red Ginseng.^9^ It has the effects of promoting pulse growth, nourishing yin and promoting fluid flow, and tonifying qi and solidifying dehydration. Studies have reported that SMI administered during chemotherapy has the effect of increasing treatment efficacy and reducing toxicity.^6^-^8^ However, most studies are in the field of lung cancer,^10^ breast cancer,^11^ and gastric cancer^12^, but few on CRC. In this study, we aimed to investigate the inflammatory response and side effects of chemotherapy combined with SMI in the treatment of patients with advanced CRC.

## METHODS

This retrospective cohort study included the clinical records of 152 patients (90 males and 62 females) with CRC admitted to the First Affiliated Hospital of Huzhou University from April 2020 to April 2023. The patients were divided into control group (patients received chemotherapy treatment, n=75) and observation group (patients received chemotherapy combined with SMI, n=77) based on the treatment received.

### Ethical Approval:

The study was approved by the ethics committee of our hospital with the number 2022118 on January 18, 2023.

### Inclusion criteria:

- Patients diagnosed as CRC by pathological examination.^13^
- Patients with a clear diagnosis of stage IIIb or IV.
- The clinical data is complete.

### Exclusion criteria:

- Existence of other benign and malignant tumors.
- Existence of organic lesions such as kidney and liver.
- Presence of intestinal bleeding, perforation, and obstruction.
- Severe malnutrition.

### Chemotherapy treatment:

All patients were administered intravenous infusion of 130 mg/m^2^ oxaliplatin on the first day (Jiangsu Hengrui Pharmaceutical Co., Ltd.; approval number: 220215BA), intravenous infusion of 200 mg/m^2^ calcium folinate on the 1st to 2nd day (Jiangsu Hengrui Pharmaceutical Co., Ltd.; approval number: 220203BL), intravenous infusion of 400 mg/m^2^ 5-fluorouracil (batch number: 210901, Southwest Pharmaceutical Co., Ltd.) on the 1st to 2nd day. Three weeks was considered one cycle, with a total of four cycles of treatment.

### Shenmai Injection:

In addition to standard chemotherapy treatment, patients in the observation group received intravenous infusion of 50 ml SMI (Dali Pharmaceutical Co., Ltd.; approval number: Z20093647) +250 ml 5% glucose injection (Jiangsu Hengrui Pharmaceutical Co., Ltd.; approval number: H3202368); Once per day for 14 consecutive days, with a 7-day break before proceeding to the second cycle; Simultaneously with chemotherapy; 21 days per cycle; A total of 4 cycles of treatment were conducted.

### Observation indicators:

- Tumor control rate. According to the RECIST standard, the treatment effect was classified as complete remission, partial remission, stability, and progression. Stability, partial remission, and complete remission were included in the tumor control rate.^14^
- Serum levels of immune function indicators such as CD3^+^, CD4^+^, CD4^+^/CD8^+^ were measured using BriCyte E6 flow cytometry (Shenzhen Mindray Biomedical Electronics Co., Ltd.)
- Inflammatory factor indicators. Serum levels of IL-6, TNF-α, IL-8 were measured using enzyme-linked immunosorbent assay; the reagent kit was purchased from Shanghai Enzyme-linked Biotechnology Co., Ltd.
- The incidence of toxic side effects such as neurotoxicity, gastrointestinal reactions, bone marrow suppression, leukopenia, vomiting and nausea.

### Statistical analysis:

It was conducted using SPSS 25.0 software (IBM Corp, Armonk, NY, USA). The normality of the data was evaluated by the Shapiro Wilk test. The data of normal distribution were represented by mean ± standard deviation, independent sample t-test was used for inter group comparison, and paired t-test was used for intra group comparison before and after treatment. The counting data were represented by the number of cases using chi square test. P-value less than 0.05 was considered statistically significant. All reported p-values were bilateral.

## RESULTS

A total of 152 patients were included. Age of the patients ranged from 45 to 81 years, with an average of 64.31 ± 7.84. Baseline data in the two groups was comparable (*P*>0.05) (Table-I). The tumor control rate in the observation group (89.61%) was higher than that in the control group (77.33%) (*P*<0.05) (Table-II).

**Table-I:** Comparison of baseline data between two groups.

Baseline data	Observation group (n=77)	Control group (n=75)	χ^2^/t	P
Gender (male/female)	44/33	46/29	0.276	0.599
Age (years)	64.97±7.54	63.63±8.13	1.060	0.291
Disease Staging			0.292	0.589
III b	46 (59.74)	48 (64.00)		
IV	31 (40.26)	27 (36.00)		
Pathological type			1.512	0.469
Squamous cell carcinoma	10 (12.99)	6 (8.00)		
Adenosquamous carcinoma	14 (18.18)	18 (24.00)		
Adenocarcinoma	53 (68.83)	51 (68.00)		

**Table-II:** Comparison of tumor control rates between two groups.

Group	n	Complete remission	Partial relief	Stabilize	Progression	Total effective rate
Observation group	77	6(7.79)	39(50.65)	24(31.17)	8(10.39)	69(89.61)
Control group	75	2(2.67)	29(38.67)	27(36.00)	17(22.67)	58(77.33)
*χ^2^*						4.167
*P*						0.041

Before treatment, there was no statistically significant difference in the levels of CD3^+^, CD4^+^, CD4^+^/CD8^+^ between the two groups (*P*>0.05). After treatment, CD3^+^, CD4^+^, CD4^+^/CD8^+^ levels in the observation group were significantly higher than those in the control group (*P*<0.05) (Table-III).

**Table-III:** Comparison of immune function between two groups.

Time	Group	n	CD3^+^	CD4^+^	CD4^+^/CD8^+^
Before treatment	Observation group	77	29.51±4.08	23.70±3.89	0.90±0.23
	Control group	75	30.19±4.27	24.09±4.07	0.95±0.25
	*t*		-1.004	-0.607	-1.105
	*P*		0.317	0.545	0.271
After treatment	Observation group	77	35.58±3.98^a^	30.90±4.19^a^	1.26±0.27^a^
	Control group	75	30.63±3.65	23.61±4.26	0.98±0.24
	*t*		7.994	10.626	6.769
	*P*		<0.001	<0.001	<0.001

***Note:*** Compared with the same group before treatment,

a P<0.05.

Before treatment, there was no statistically significant difference in the levels of IL-6, TNF-α, and IL-8 between the two groups (*P*>0.05). After treatment, serum levels of IL-6, TNF-α, and IL-8 in both groups significantly decreased compared to before treatment, and were significantly lower the observation group compared to the control group (*P*<0.05) (Table-IV).

**Table-IV:** Comparison of inflammatory factor index levels between two groups (ng/L).

Time	Group	n	IL-6	TNF-α	IL-8
Before treatment	Observation group	77	15.40±2.66	208.09±32.29	250.56±35.74
	Control group	75	14.52±3.26	213.57±33.49	252.65±36.15
	*t*		1.812	-1.028	-0.359
	*P*		0.072	0.306	0.720
After treatment	Observation group	77	7.29±2.55^a^	87.81±14.80^a^	115.49±21.41^a^
	Control group	75	9.05±3.15^a^	111.41±23.03^a^	151.81±34.39^a^
	*t*		-3.776	-7.539	-7.838
	*P*		<0.001	<0.001	<0.001

***Note:*** Compared with the same group before treatment,

a P<0.05.

The incidence of neurotoxicity (20.78%), gastrointestinal reactions (24.68%), bone marrow suppression (29.87%), leukopenia (33.77%), vomiting and nausea (35.06%) in the observation group was lower than that in the control group (46.67%, 49.33%, 45.33%, 52.00%, 56.00%, respectively) (*P*<0.05) (Table-V).

**Table-V:** Comparison of the incidence of toxic and side effects between two groups.

Group	n	Neurotoxicity	Gastrointestinal reactions	Bone marrow suppression	Leukopenia	Vomiting and nausea
Observation group	77	16(20.78)	19(24.68)	23(29.87)	26(33.77)	27(35.06)
Control group	75	35(46.67)	37(49.33)	34(45.33)	39(52.00)	42(56.00)
*χ* ^2^		11.420	9.928	3.876	5.161	6.718
*P*		0.001	0.002	0.049	0.023	0.010

## DISCUSSION

The results of this study showed that chemotherapy combined with SMI can significantly enhance the immune function of patients with CRC, improve the treatment effect of the disease, and reduce the occurrence of toxic side effects compared to chemotherapy alone. The findings of this study is consistent with a bayesian network meta-analysis by Liu et al.^15^

Traditional Chinese medicine categorizes colorectal cancer into categories such as “bloody stool”, “intestinal wind”, and “colon”. Its onset is caused by toxic pathogens damaging collaterals, phlegm and blood stasis coagulation, and chemotherapy is a heat toxic pathogen. Fire heat damages yin, and both resistance and immune function are damaged, resulting in fullness, bloating, and fatigue.

SMI contains extracted Ophiopogon japonicus and Red Ginseng, which can generate pulse, tonify qi and solidify dehydration, nourish yin and promote fluid production. The effective ingredients of Red Ginseng have biphasic immune regulation function, and can improve specific and non-specific immune function. The polysaccharides in Ophiopogon japonicus can promote cellular and humoral immune functions.^16^,^17^ Cheng L et al.^18^ confirmed that SMI can lower the degree of toxic side effects caused by chemotherapy, strengthen the body’s immune function, enhance the killing ability of natural killer cells against lesion cells, and achieve the goal of increasing efficacy and reducing toxicity, which is consistent with the results of our study. Chen Y et al^19^ discovered that SMI can promote tumor cell apoptosis in lung adenocarcinoma, inhibit gene expression of nuclear proliferation antigen gene, prevent the formation of micro-vessels in tumor tissue, and thus prevent tumor growth. Zhong C et al^20^ confirmed that SMI can exert synergistic anti angiogenic effects when combined with chemotherapy. It has the ability to prevent proliferation and migration of microvascular endothelial cells in vitro, and can synergistically inhibit angiogenesis in vivo. The meta-analysis by Qin GW et al.^21^ that focused on the combination of SMI and chemotherapy regimen for the treatment of lung cancer confirmed that adding SMI can enhance the body’s immune function, improve short-term efficacy of the treatment, improve patient quality of life, and reduce adverse reactions caused by chemotherapy drugs. Our conclusion is consistent with this research.

Kasprzak et al^22^ confirmed that TNF-α is often abnormally highly expressed in CRC patients. As a pro-inflammatory factor, TNF-α can promote the onset and progression of tumors.^23^ IL-6 and IL-8 are both important factors closely related to the onset and progression of colorectal cancer.^24^,^25^ IL-6 can accelerate tumor cell proliferation and growth, affect immune cell function, and promote the progression of CRC.^26^ IL-8 has functions such as regulating vascular permeability and proliferation, and promoting tumor vascular proliferation. Specifically, it increases the number of local neutrophils within the tumor microenvironment, and accelerates tumor metastasis and infiltration.^26^,^27^ The results of our study showed that the serum levels of IL-6, TNF-α, and IL-8 in the observation group were lower than those in the control group after the treatment, indicating that chemotherapy combined with SMI can effectively alleviate inflammatory reactions in patients with advanced CRC. We propose a synergistic mechanism, where chemotherapy can kill tumor cells and inhibit lesion progression, while the addition of SMI can enhance patient’s immune function and improve the disease treatment effect. Therefore, the combined regimen can more effectively downregulate the expression of inflammatory factors and reduce the degree of inflammatory response.

### Limitations:

Firstly, this is a single center retrospective analysis with a small sample size and selection bias. Secondly, there are few observation indicators. Finally, we did not conduct a long-term follow-up. Further studies with longer follow-ups on the patient’s quality of life and long-term prognosis are needed.

## CONCLUSION

Chemotherapy combined with SMI can better alleviate inflammatory response, enhance immune function, and improve tumor control rate in patients with advanced colorectal cancer compared with chemotherapy alone. The combined regimen is associated with a lower incidence of toxic side effects.

### Authors’ contributions:

**YJ:** Conceived and designed the study. **YJ**, **WZ**, **HD** and **XH:** Collected the data and performed the analysis. **YJ:** Was involved in the writing of the manuscript and is responsible for the integrity of the study. All authors have read and approved the final manuscript.
